# Drunken Environments: A Survey of Bartenders Working in Pubs, Bars and Nightclubs

**DOI:** 10.3390/ijerph10104896

**Published:** 2013-10-11

**Authors:** Sébastien Tutenges, Trine Bøgkjær, Maj Witte, Morten Hesse

**Affiliations:** Centre for Alcohol and Drug Research, Aarhus University, Artillerivej 90, Copenhagen 2300, Denmark; E-Mails: stu@crf.au.dk (S.T.); trinebogkjar@gmail.com (T.B.); maj_witte@hotmail.com (M.W.)

**Keywords:** alcohol, violence, stress, drinking environments, prevention, work, bartenders

## Abstract

There is evidence that bartenders play a key role in respect of the health and safety of patrons in nightlife environments. However, little is known of how bartenders themselves are affected by the environments in which they work, especially with regard to their exposure to violence, pressure to drink and stress. We used a cross-sectional survey to assess the experiences of bartenders (n = 424) working in pubs, bars and nightclubs in Denmark. 71% of the respondents reported drinking while working, 6% reported using other drugs than alcohol at work, and 33% reported drinking even when they did not feel like it because of pressure to drink at work. Verbal assaults and threats were common and associated with higher levels of perceived stress. Bartenders’ work environment poses a risk for the development of alcohol use disorders. The fact that many bartenders consume significant quantities of alcohol during their working hours may pose a risk not only to their own safety, but also to that of their colleagues and patrons.

## 1. Introduction

A substantial proportion of at-risk drinking takes place in nightlife environments such as bars, pubs and nightclubs. For example, a recent UK study showed that 72% of males would reach a blood-alcohol concentration of 0.15 during a night out [1]. The high levels of alcohol consumption in nightlife environments carry other risks, including traffic risks [2] and violence [3].

Bartenders play a key role in the overall health and safety conditions in nightlife environments. They administer alcohol to patrons and, along with other staff such as bouncers and DJs, contribute to the regulation of those environments. Environmental health research on bartenders has so far focused mainly on exposure to cigarette smoke and subsequent risks of respiratory and cardiovascular disease, a problem addressed by recent legislation against indoor smoking in many countries which has led to improved health status for nightlife workers [4,5,6]. There is also evidence that individuals working as bartenders, at least those doing so on a casual basis while travelling, frequently engage in unprotected sex, which may contribute to the spread of sexually transmitted diseases [7].

Bartenders also play a key role in patrons’ level of alcohol consumption. Despite legislation against serving to intoxicated customers and minors in many countries, several studies have found that bartenders will serve alcohol to visibly intoxicated patrons [8,9,10] and to minors [11]. Several studies suggest that the attitude of venue staff, including bartenders, is a significant predictor of the risk behaviours among patrons [12]. Unfriendly, confrontational and aggressive staff can set the tone in bars, pubs and nightclubs and incite patrons to engage in violence with staff and/or other patrons [13,14]. Studies show that training bartenders in “responsible serving” can temporarily reduce rowdiness and heavy drinking without negatively affecting the positive atmosphere at bars (e.g., [15,16]), although the outcomes vary significantly between studies [17].

Relatively little research, however, has been conducted on the broader spectrum of health risks to which bartenders are or may be exposed. There is some evidence that, in addition to the increased risk of tobacco-related mortality, bartenders are also at higher risk of dying from alcohol-related disease and homicide [18]. Findings such as these suggest that general lifestyle factors, work-related variables, or a combination of both, may lead to severe health problems among bartenders.

The question of alcohol use among bartenders is significant, then, in light of the fact that heavy alcohol use at work may put venue staff at risk of both short- and long-term health damage, and may also impede the staff’s ability to prevent harm among patrons or nuisance in the local community.

The aim of this study was to assess risk factors in the working environments of bartenders in four cities in Denmark. The cities were selected to represent both urban and provincial areas and high income and low income areas.

The study aim included focus on the following areas:

- Exposure to patron aggression at work
- Stress
- Alcohol and other drug use at and away from work

## 2. Methods

This study is based on data from a cross-sectional survey conducted in four cities in Denmark. Questionnaires were administered to bartenders working at nightlife venues catering for patrons aged between 15 and 35 years. The venues chosen included a wide variety of establishments, including what may be termed discotheques/nightclubs, live music venues, traditional pubs, combination of traditional pub and discotheque and café/bar. Two research assistants collected the data over a period of seven months, from June to December 2012. 

### 2.1. Procedures

Three different strategies were employed to get in contact with the bartenders: (1) The primary strategy used was to attend staff meetings at the targeted nightlife venues. Research assistants informed the bartenders about the research project at the meeting and handed out survey questionnaires to those who agreed to participate. We could observe that alcohol was often served to the bartenders during the meetings. (2) When there was no opportunity to attend a staff meeting, research assistants obtained the bartenders’ email addresses through their employers and sent an information email containing a link to an online version of the questionnaire for them to fill out if they wished to participate. This strategy was also employed to obtain replies from bartenders absent at the staff meetings. (3) Finally, some bartenders were contacted in their workplaces and asked by the assistants to fill out printed questionnaires while at work.

Before the bartenders were asked to participate they were informed about the institutional context and aim of the study, the length of the questionnaire and their status of anonymity. In instances where the contact with the bartenders was mediated through their employer, the research assistants emphasised that participation in the survey was voluntary and that it was not a work-related assignment.

Any instances of employers declining to communicate contact to their employed bartenders were recorded, together with their reason for refusal. Any instances of individual bartenders declining to participate after having been informed about the project were also recorded.

### 2.2. Participants

Sixty one nightlife venues were contacted for inclusion in the study. These venues were identified after consultation with local police, licensing staff and other key stakeholders in the night-time economy. The managers or owners in three of the selected venues declined to collaborate in the study. Accordingly, we administered questionnaires to bartenders working for 58 different venues in the four selected cities.

A total of 489 bartenders were approached, of whom 65 refrained from participating in the survey after having been informed about the research project. Thus the overall response rate for the survey is 86.71%. The survey questionnaire took approximately 10 min to complete. The questionnaire included questions on background information such as gender, age, birth country of both parents and level of education. It also explored the bartenders’ work experience, including the type of bars that they worked in (see above), whether bartending was their main or secondary occupation, the number of years they had been bartending and the number of night shifts that they had worked as a bartender during the past 12 months.

### 2.3. Type and Size of Venue

Type of venue was determined by asking: “Which description best fits the place where you bartend the most?” Response options were: “Discotheque/nightclub”, “Live music venue”, “Traditional pub”, “Combination of traditional pub and discotheque” and “Café/bar”. Respondents were also asked what the maximum capacity of the venue was, with response categories “less than 50”, “50 to 99”, “100 to 199”, “200 to 299” or “300 or more”. 

### 2.4. Violent Work Environment

Violent work environment was assessed using the following questionnaire items, each referring to the past year and to work-related incidents only: (a) “How often have you been yelled at with foul language?” (b) “How often have you been verbally threatened?” (c) “How often have you been threatened with a weapon or something similar to a weapon?” (d) “How often have you been spat at, hit, kicked, had objects thrown at you, or similarly attacked by guests or others?” (e) “How often have you seen your colleagues exposed to violence?” (f) “How often have you had to physically intervene in a violent conflict?” and (g) “How often have you seen an assault, a fight or something similar?” Six response options were given, ranging from “not in the past twelve months” to “once or more per week”.

A factor analysis was conducted to test whether these items loaded on a single factor. As the items were measured on an ordinal scale, polychoric correlations were used to assess a principal components analysis. A single factor explained 73% of the variance, with an Eigenvalue of 5.10, and the second factor accounted for 8% of the variance and had an Eigenvalue of 0.55. Based on parallel analysis, a single factor model was preferred. Therefore, the violence items were combined to form a single scale (Cronbach’s alpha = 0.87).

### 2.5. Stress

Stress was measured using the following items: (a) “How often have you felt stressed in the past twelve months?” (b) “How bad was the worst stress you have had in the past year”, (c) “How often have you had trouble sleeping in the past 12 months?” and (d) “How bad were the worst sleeping problems you have had in the past year?” (Various response options were used, including: daily, weekly, monthly, never/less than monthly). Again, a factor analysis was conducted to test whether these items loaded on a single factor, using polychoric correlations. A single factor explained 67% of the variance, with an Eigenvalue of 2.67, and the second factor accounted for 24% of the variance and had an Eigenvalue of 0.98. Based on parallel analysis, a single factor model was preferred. Therefore, the stress items were combined to form a single scale (Cronbach’s alpha = 0.78).

### 2.6. Alcohol and Other Drug Use

Alcohol and other drug use were measured using the following items: (a) “On how many occasions in the past month did you drink five or more drinks in one sitting?” (b) “On how many occasions in the past month did you drink five or more drinks while at work?” (c) “On how many occasions in the past month did you drink ten or more drinks while at work?” (d) “How many times did you use *substance* in the past year?” Nine response options were provided for these items, from “never” to “nearly daily”. The following question was also asked: “How many times did you use *substance* in the past year while working?” The substances were alcohol, cannabis, ketamine, ecstasy, khat, amphetamine, Ritalin, magic mushrooms, steroids and other.

For alcohol, we asked about the respondents’ total alcohol consumption over an average week. The question asked was “How many units do you drink on average per week?” with a unit defined as “one beer, one glass of wine or 4 cL of strong liquor”.

### 2.7. Statistical Analyses

We report descriptive statistics. For analyses of stress and violence the dependent variables were highly skewed, and we used rank-transformed variables for regression analyses. For frequency of drinking five or more drinks at work we used negative binomial regression analysis, as the data did not fit a Poisson model. We used two dummy variables for pressure to drink (“Often” *versus* “Never”, and “Sometimes” *versus* “Never”), and total number of shifts in the past month as an exposure variable, since only shifts would be occasions to drink at work.

## 3. Results

The sample comprised 199 women and 225 men. The average age was 26.38 (standard deviation (SD) = 6.13, range 17 to 69 years). Of the respondents, 12.06% had fewer than 10 years of school, 44.44% had a high-school education, 16.55% were trained in some skill or trade (e.g., policeman, carpenter or painter), 15.84% had a BA and 11.11% had an MA or equivalent. 

The respondents had worked on average for 2.71 years as a bartender (SD = 3.35). The median was 2.0, and the most experienced respondent had worked for 30 years as a bartender. The average number of night shifts in the past month was 7.36 (SD = 6.03, range 0 to 28).

Most respondents worked in a “Discotheque/nightclub” (45.73%), followed by a “Combination of traditional pub and discotheque” (19.10%), “Café/bar” (18.09%), “Live music venue” (12.31%) and “Traditional pub” (4.77%). Almost half of the respondents worked in venues with a maximum capacity of 300 or more guests (45.05%), 16.75% worked in venues with room for 200 to 299 guests, 16.75% in venues with room for 100 to 199 guests, 14.39% in venues with room for 50 to 99 guests and 3.54% in venues with room for fewer than 50 guests. 3.54% did not answer this question.

### 3.1. Violence

The violence experienced while working in the nightlife environment is summarised in Table 1. The majority of respondents had experienced being yelled at with profanities or witnessing violence more than once in the past year. Threats with weapons and direct assaults were the rarest types of violence experienced. The combined violence scale was heavily skewed and had a high kurtosis (skew = 1.94, kurtosis = 8.18). For this reason, Spearman rank-order correlations are reported between violence and other variables. Experiencing more violence was weakly associated with age (*ρ* = −0.09, *p* = 0.07), but no age difference was observed (Mann-Whitney test *z* = −0.29, *p* = 0.77). In contrast, experiencing more violence was moderately associated with working more shifts in the past month (*ρ* = 0.26, *p* < 0.001), working more months in the past year (*ρ* = 0.34, *p <* 0.001), and working more years in the nightlife environment (*ρ* = 0.17, *p <* 0.001).

**Table 1:** Descriptive statistics for violent events while bartending.

	Never	Once in past year	More than once	Valid N	Item-rest correlation
Been yelled at	30.41%	12.90%	56.69%	411	0.66
Threatened verbally	61.07%	13.63%	25.30%	411	0.74
Threated with weapon	95.60%	2.44%	1.96%	409	0.56
Attacked	82.89%	9.54%	7.57%	409	0.69
Witnessed assault on colleague	74.26%	12.01%	13.73%	408	0.70
Had to intervene	64.95%	13.48%	21.57%	411	0.69
Witnessed violence	29.90%	14.22%	55.88%	411	0.65

### 3.2. Stress

Of all respondents, 48.73% reported never feeling stressed, 33.71% monthly, 13.60% weekly, and 3.97% daily, while 46.72% reported never having problems sleeping, 26.51% reported monthly, 20.47% weekly, and 6.30% daily. We regressed the aggregated scale on degree of violence experienced in the past month, number of shifts worked in the past month, months working as a bartender in the past year, gender, and age. Degree of violence experienced was associated with higher degree of stress (*r_p_* = 0.22, *p <* 0.001). In addition, women reported higher levels of stress (*r_p_* = 0.14, *p <* 0.01), and bartenders who had worked more shifts in the past month reported more stress (*r_p_* = 0.10, *p* = 0.04). The results of the regression analysis are shown in Table 2.

**Table 2:** Predictors of higher scores on stress.

	Raw correlation	Partial correlation	T-score	*p*-value
Experiencing violence at work	0.24	0.20	4.08	0.000
Months working as bartender	0.03	−0.07	−1.45	0.148
Shifts in past month	0.15	0.10	1.98	0.048
Gender	0.14	0.14	2.79	0.005
Age	−0.08	−0.00	−0.59	0.557

### 3.3. Alcohol and Other Drug Use

Of the sample, 97.56% reported using alcohol in the past year. The men in our sample reported drinking 18.50 drinks on a typical week (SD = 14.43), and the women 11.74 (SD = 12.18). 28.86% drank in excess of the recommended limit for unhealthy intake stipulated in the Danish National Board of Health safe drinking guidelines (seven units for women and 14 for men is defined as the limit for unhealthy drinking) [19]. 

Of all respondents, 68.3% reported drinking alcohol on a normal shift, and 40.15% reported drinking more than five drinks on at least one shift in the past month. In addition, 17.57% reported drinking 10 or more drinks during at least one shift in the past month.

When asked about whether they would drink at work when not feeling like it, 28.47% reported occasionally doing so and 4.21% reported doing so often.

In a negative binomial regression analysis, sometimes feeling pressured to drink at work was associated with a higher number of drinks on a typical shift (*beta* = 0.55, *z* = 2.57, *p* = 0.010), and often feeling pressured was additionally associated with a higher level of drinking (*beta* = 1.32, *z* = 2.62, *p* = 0.009). Low frequency of drinking 5+ at work was associated with female gender (*beta* = −1.03, *z* = 5.03, *p <* 0.001) and older age (*beta* = −0.05, *z* = −2.60, *p* = 0.009).

39.11% of respondents reported using cannabis in the past year, 12.31% cocaine, 8.33% ecstasy, 5.79% amphetamine, 4.55% Ritalin, 4.02% ketamine, 4.04% magic mushrooms, 2.53% benzodiazepines and 2.02% steroids. Any drug use was reported by 44.10% (59.11% of men and 27.14% of women). In total, 5.00% reported using cannabis while working. No other drugs were reported during work by any more than 1% of the respondents. Men were more likely than women to report use of all drugs including cannabis (54.03% *versus* 22.80%, *χ*^2^(1) = 41.28, *p <* 0.001), cocaine (24.89% *versus* 9.55%, *χ*^2^(1) = 17.07, *p <* 0.001), amphetamine (8.00% *versus* 2.51%, *χ*^2^(1) = 6.20, *p <* 0.013) and methylphenidate (7.56% *versus* 0.50%, *χ*^2^(1) = 12.92, *p <* 0.001).

### 3.4. Inter-Correlations of Stress, Violence and Alcohol Consumption

As noted above, stress and violence in the work environment were correlated. However, number of alcoholic drinks consumed on a typical shift was not associated with stress (*ρ* = −0.09, *p* = 0.07) or violence at work (*ρ* = −0.03, *p* = 0.59). No significant differences were found with regard to stress or experiences of violence between drug users and non-users.

## 4. Discussion

In this study, we found that drinking alcohol at work was common among bartenders, and that a substantial proportion of the bartenders reported drinking heavily while working at least once during the last month. We also found that a large proportion experienced violence at work, and that violence was associated with higher levels of stress. We did not find that use of illicit drugs was common behind the bar: of our respondents, 5.00% reported smoking cannabis on shifts, and other drug use was very uncommon.

Certainly, not every instance of alcohol use at work represents a serious problem. A person having one or two drinks at the end of a shift may not be at any particular risk, and consuming five units over an eight-hour shift may not lead to intoxication. However, there is ample evidence that recalled use of alcohol represents a low estimate of alcohol consumption (e.g., [20]), especially when respondents have been consuming relatively large amounts of alcohol [21]. This study does not indicate that every bartender is on the road to alcoholism, but rather that a significant proportion of bartenders drink in ways that are associated with risks of short- and long-term harm.

One potential consequence of alcohol consumption at work for bartenders is unintentional injury, particularly since handling glass and carrying heavy items such as boxes of beer and beer kegs is a continual part of their work.

With regard to substances other than alcohol, 44.10% reporting illicit drug use is a high proportion compared with the most recent general population survey from Denmark, which showed that 23.9% of men and 16.1% of women in the age bracket from 16 to 24 years reported past year cannabis use, and lower frequencies for older age groups ([22], Chapter 5.7). Thus, there is some indication that bartenders are more likely to be drug users compared with the rest of the Danish population.

Our study also identified a number of other risk factors in bartenders’ work environment. While at work, bartenders are exposed to a substantial risk of violence, and one in three reported feeling that they had to drink while at work. In addition, those who reported feeling pressured to drink at work consumed significantly more alcohol on a typical shift.

Being exposed to high levels of violence in the form of threats and fights during shifts was associated with higher levels of stress. This is not surprising: violent confrontations are largely uncontrollable situations that provoke tension, nervousness or paralysing fear in most humans, and there is a real danger that the confrontations lead to physical and psychological harm [23]. Moreover, in some respects bartenders are in a vulnerable situation when conflicts erupt: they may not be able to leave the bar freely, they often have to return to the scene of confrontation (their workplace) and they often have no training in managing severe confrontational situations. Stress is known to carry long-term risks of both physical disease and mental disorders (e.g. [24,25]), and exposure to uncontrollable stressors may negatively affect long-term ability to cope with stress [26].

The violence experienced at work was associated with higher levels of perceived stress, but not with drinking or drug use. However, the combination of violence and bartenders’ being under the influence of alcohol poses risks of its own. For instance, many of the bartenders reported being threatened or having to intervene in physical confrontations. In such situations, bartenders who are under the influence of alcohol may misinterpret situations and escalate conflicts or fail to intervene appropriately. Moreover, if bartenders are to handle responsible serving and not serve underage or intoxicated costumers, having consumed alcohol is detrimental to consistent performance.

It is interesting that those who had worked longer in nightlife venues reported more violence, as it might be expected that their experience would enable them to reduce their exposure. We are not certain what lies behind this correlation. It may be a spurious correlation, but it is also possible that bars with a rougher clientele would tend to employ more experienced bartenders and that inexperienced bartenders would quickly stop working at these bars, leading to a survival bias.

### 4.1. Implications for Policy

The findings indicate that the short- and long-term health of bartenders may be improved by focusing more on alcohol consumption behind the bar. There is currently no legislation prohibiting or regulating bartender’s alcohol consumption in Denmark. It is left to the discretion of individual venues to decide whether or not their bartenders can drink while working, but many venues only have vague or non-existing alcohol policies for their employees. A combination of legislation and collaboration between venue owners and local authorities may be a way to reduce the extent of alcohol use among bartenders. This may also pave the way for engaging bartenders in reducing violence and conflicts in venues, for instance by enforcing existing laws prohibiting the serving of alcohol to intoxicated or under-age patrons [15].

### 4.2. Limitations and Strengths

To the best of our knowledge this is the first study to assess bartenders’ working environments in relation to violence, stress and pressure to drink. Our assessment was limited to the focus points of our study, and did not fully cover the working conditions of bartenders in pubs, bars and nightclubs.

The cross-sectional nature of our study limits the interpretation of the findings. We cannot rule out, for instance, the possibility that the association between stress and experiencing violence may be due in part to the response style of some of our respondents, or memory bias such that those with higher levels of stress were more likely to remember unpleasant events. The fact that alcohol was often consumed at the staff meetings may also have affected the respondents’ answers.

Although we had an acceptable response rate, higher than 80%, we are also likely to have missed certain groups of bartenders. In particular, non-Danish speaking bartenders were not able to respond to our questionnaire. From our contact with bartenders and venue owners we have observed that a large number of people who have recently arrived in Denmark work as bartenders.

Further, our sample was not random, and included only venues in areas of the city with a high density of venues and where there is a large amount of nightlife activity on weekend evenings. Venues in the suburbs were not covered, including traditional pubs outside of the city centre that cater for locals who may drink in a manner that is more frequent but less focused on states of deep intoxication.

## 5. Conclusions

Some bartenders consume a large amount of alcohol. A high level of exposure to violence, high consumption of alcohol and a pressure to drink alcohol all constitute risk factors for physical and mental health, and contribute to a poorly regulated nightlife. Strategies to regulate alcohol in the work environment of bartenders could benefit bartenders and provide one central precondition for the implementation of safer nightlife practices.
